# Fabrication of an Optical Sensor Based on Eosin-Y-Doped Electrospun Fibers for Ammonia Detection via Wavelength Shifts

**DOI:** 10.3390/nano15040273

**Published:** 2025-02-11

**Authors:** Rispandi, Manna Septriani Simanjuntak, Cheng-Shane Chu

**Affiliations:** 1 Department of Mechanical Engineering, Faculty of Engineering, Universitas Andalas, Padang 25163, West Sumatera, Indonesia; rispandi@eng.unand.ac.id; 2 Department of Mechanical Engineering, Ming Chi University of Technology, Taishan District, New Taipei City 24301, Taiwan; mannasimanjuntak12@gmail.com; 3 International Ph.D. Program in Innovative Technology of Biomedical Engineering and Medical Devices, Ming Chi University of Technology, New Taipei City 24301, Taiwan; 4 Research Center for Intelligent Medical Device, Ming Chi University of Technology, Taishan District, New Taipei City 24301, Taiwan

**Keywords:** optical sensing, electrospinning, ammonia, Eosin-Y, cellulose acetate

## Abstract

This research presents a simple and effective technique to fabricate an optical sensor for ammonia detection, leveraging emission wavelength shifts as the sensing mechanism. The sensor comprises a cellulose acetate matrix doped with Eosin-Y, which serves as the electrospinning material. Photoluminescent micro/nanofibers were successfully fabricated using electrospinning and were stimulated by a 380 nm central wavelength LED. The Eosin-Y-doped electrospun fiber membranes exhibited a red emission peak at 580 nm, allowing ammonia to be detected in the linear concentration range of 0–500 ppm. The experimental results demonstrated a high sensitivity of 8.11, with a wavelength shift sensitivity of 0.029 nm/ppm in response to ammonia concentration changes. This optical sensing method effectively mitigates the influence of fluctuations in excitation light intensity, offering improved reliability. The Eosin-Y-containing electrospun fibers show great potential as a practical sensing material for detecting ammonia gas concentrations with high precision, supporting diverse applications in medical diagnostics, environmental monitoring, and industrial processes.

## 1. Introduction

Gas detection has become essential to ensure optimal conditions and provide early warnings of potential hazards to human health and safety in industrial and medical applications [1,2]. Researchers have made significant advancements in developing sensors across various domains, including environmental monitoring, manufacturing, medical applications, and indoor air quality management [3]. Gas sensors are specifically designed to identify the presence of gas mixtures in hazardous environments, where they play a critical role in protecting humans and animals [4]. Detecting toxic gases like ammonia is crucial in various fields, industries, laboratories, environmental, and medical applications [5]. The global economic impact of advanced manufacturing sectors, including Additive Manufacturing (AM), commonly known as 3D printing, has significantly transformed production processes by enabling the creation of complex designs with reduced material waste and shorter lead times. This transformation has led to substantial economic benefits across various industries. For instance, the global market for AM is expected to grow, which may increase the prominence of sustainability aspects in the manufacturing process [6]. Detecting toxic gases, such as ammonia, is crucial for maintaining safety in industries, laboratories, food storage, health monitoring, and safety applications. The global gas detection equipment market was valued at approximately USD 5.60 billion in 2024 and is projected to grow at a compound annual growth rate (CAGR) of 11.7% from 2025 to 2030. This growth is driven by stringent safety regulations and the increasing demand for real-time monitoring solutions [7]. Specifically, the market for fixed ammonia gas detectors was valued at approximately USD 560 million in 2023 and is projected to reach nearly USD 1.02 billion by 2032, growing at a CAGR of 6.9% during the forecast period. This underscores the critical importance of ammonia detection in ensuring safety and compliance across various sectors [8]. Collectively, advancements in manufacturing technologies and the emphasis on safety through effective gas detection systems are contributing to significant economic impacts globally. With increasing environmental and human health concerns, NH_3_ gas detection has significantly increased attention in sensor development. Ammonia is one of the most extensively produced and used chemicals in the world [9,10,11,12,13,14,15]. Because ammonia can be dangerous even at low concentrations, the sensors made to detect it need to be extremely sensitive and quick to react [16].

The effectiveness of a sensor is determined by key parameters such as sensitivity, selectivity, stability, and recovery [17,18]. Fluorophore-based sensors usually have high sensitivity [19]. Among the various types of optical sensors, fluorescent sensors are the most commonly employed. Table 1 summarizes the optical properties of materials used for ammonia detection. Prior research has investigated single- and double-layer techniques to enhance linearity and fluorescence intensity [20]. Additionally, the incorporation of fluorescent dyes into polymer matrices has been found to enhance sensitivity, stability, and response time [21]. The advantageous properties of micro- and nanofiber membranes, such as their high porosity and huge surface area, are primarily responsible for this development [22,23]. Because of its affordability, adaptability, ease of use, and sophisticated capabilities, electrospinning has gained popularity as a method for creating nanomaterials. This method employs electrical forces to generate fibers from materials such as polymers, ceramics, or graphite, forming a structure known as the Taylor cone. Numerous factors, such as flow rate, applied voltage, distance, viscosity, conductivity, and the makeup of the polymer solution, affect the characteristics of the final electrospun fibers [24]. Each of these elements is essential in determining the fibers’ surface shape as the Taylor cone forms. By fine-tuning these parameters, it is possible to fabricate nanofibers with precisely tailored surface characteristics [25].

A scanning electron microscope (SEM) will be used to investigate the morphology of the manufactured membrane fibers in order to determine the structure and properties of each individual fiber. This study introduces a novel, straightforward ammonia-sensing technique based on single-fiber wavelength shifts and fluorescence quenching. To act as an ammonia indicator, Eosin-Y is embedded in cellulose acetate, and electrospinning is used in the optical sensing procedure. The fibers produced through electrospinning are subsequently tested for their ammonia gas-sensing capabilities [26].

The electrospun fibers developed in this research demonstrate effective ammonia detection, making them suitable for practical applications across various fields, environmental monitoring, medical, and industrial processes. Finally, other alternative detection platforms are based on bicolor fluorescence. These sensors exhibit high selectivity and sensitivity. However, their practical application is hindered by a high level of structural and operational complexity, particularly in designing stable supramolecular architectures. Additionally, successive runs reveal performance degradation due to photobleaching, irreversible adsorption at interfaces, and environmental susceptibility, limiting their long-term reliability in experimental conditions [27]. The electrochemical sensors underscore their significant role in various analytical fields due to their high sensitivity, selectivity, and rapid response times. However, their practical deployment is often constrained by a high level of complexity in sensor fabrication, calibration, and signal interpretation, particularly for multi-analyte detection. Furthermore, successive runs frequently lead to performance degradation caused by electrode fouling, material instability, and signal drift, necessitating frequent recalibration and maintenance to ensure consistent accuracy and reliability [28].

**Table 1:** Properties of materials used in optical ammonia sensors for detecting typical gases.

NH3 Probe	Method/Type	Range	Sensitivity/Linearity	Sensing Analysis	Reference
TMOF 6 (Cl)	Deposition/drop quartz plate	0–400 ppm	5/Linear	Intensity	[29]
CsPbBr_3_–SiO_2_	Drop coating	2160–3600 ppm	0.8/Linear	Intensity	[30]
ZnO: Eu^2+^	Spin coating	0–80 ppm	0.05/Linear	Intensity	[31]
MAPbBr_3_-TBA	Spin coating	0–100 ppm	2.5/Linear	Intensity	[32]
Mp-TiO_2_-based MAPbBr_3_	Spin coating	0–100 ppm	9/Linear	Intensity	[33]
CsPbBr_3_ QDs	Electrospinning	0–30 mg/L	0.8/Linear	Intensity	[34]
CsPbBr_3_ QDs	Electrospinning	0–350 ppm	0.2/Linear	Intensity	[35]
MMPyP and TMPyP	Dip coating	0–600 ppm	None/None	Intensity	[36]
CdSe/SiO_2_	Dip coating	0–400 ppm	42.4/Linear	Intensity	[37]
Eosin-Y	Spin coating	0–1000 ppm	4.8/Non-linear	Intensity	[38]
Eosin-Y	Spin coating	0–1000 ppm	20/Non-linear	Intensity	[39]
Eosin-Y	Liquid fluxion coating	0–500 ppm	None/None	Intensity	[40]
Eosin-Y	Electrospinning	0–500 ppm	8.11/Linear	Intensity/Wavelength Shift	This work

## 2. Experiments

### 2.1. Materials

A material is defined as a substance or a combination of components that make up an object. This study utilized solutes (polymers) and solvents, with the addition of fluorescent dyes. Every material was utilized precisely as it was, without any additional purification. The primary ingredient used was powdered cellulose acetate (CA) purchased from Showa Chemicals in Tokyo, Japan. As solvents, acetone and dimethylacetamide (DMAc) were provided by Thermo Fisher Scientific (Heysham, UK) and Arison CO. (Hsinchu, Taiwan), respectively. The 99% pure Eosin-Y supplier was Sigma Aldrich (St. Louis, MO, USA).

### 2.2. Experimental of Ammonia Sensing

First, 1.25 g of cellulose acetate powder was dissolved in a 2:1 solution of 6 mL acetone and 2 mL DMAc to create an ammonia-sensitive solution. For an hour, the solution was swirled at room temperature. After that, 0.036 mg of Eosin-Y was added, and for 30 min, it was evenly distributed using an ultrasonic machine. To guarantee homogeneity, the solution was agitated once more at room temperature for an additional hour after ultrasonic treatment. Ultimately, fibers were produced by electrospinning the prepared solution.

**Figure 1:**
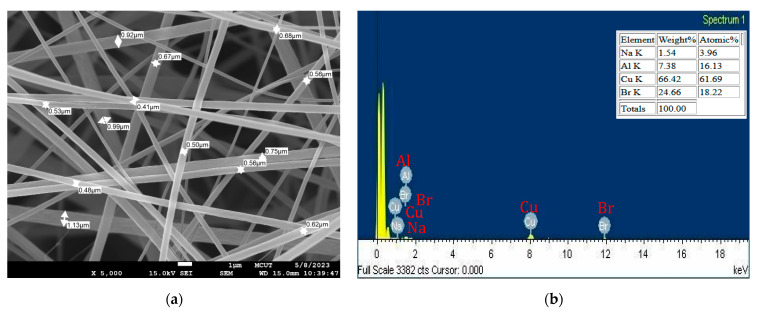
Electrospun fibers are characterized by (**a**) a SEM image with a magnification of 5000× and (**b**) an EDX spectrum analysis.

The ammonia-sensitive dye was added to an electrospinning machine to create the fibers. Scanning electron microscopy (SEM) pictures of the resultant fibers are shown in Figure 1a,b. One fiber was coated with ammonia-sensing dye and subjected to energy-dispersive X-ray spectroscopy (EDX) examination. The EDX spectrum revealed the elements present in the fiber. SEM measurements were performed at a magnification of 5000×, showing the morphology of individual fibers. Elements in a sample analyzed using Scanning Electron Microscopy (SEM) (JEOL, JEM-6701, Tokyo, Japan) with Energy Dispersive X-ray Spectroscopy (EDS or EDX) can be represented in both weight percent (Wt%) and atomic percent (At%) in the elemental composition results. Wt% represents the proportion of an element’s mass relative to the total mass of all detected elements in the sample. Meanwhile, At% represents the proportion of an element’s atoms relative to the total number of atoms of all elements in the sample. The SEM images confirm that the fibers exhibit a uniform structure with no droplet formation. To assess any aggregation effects, the experimental study on ammonia sensing evaluates potential changes in sensor response due to particle clustering or film instability, which could impact sensitivity and reproducibility. The thickness of the fiber membrane Eosin-Y-Doped Electrospun is 0.1 mm.

### 2.3. Optical Sensing Instruments

**Figure 2:**
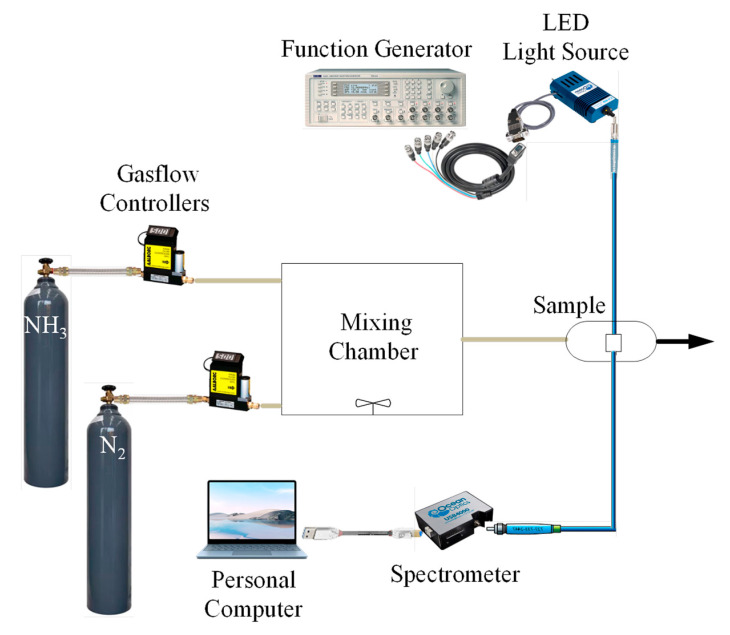
A schematic illustration of the experimental setup designed for ammonia sensing.

Figure 2 shows the arrangement for assessing the optical ammonia sensor’s performance. The system utilizes an absorption spectrophotometer integrated with a single-fiber sensor to measure the absorption spectrum of light components. By stimulating the optical sensor with an LED light source that shines at a peak wavelength of 380 nm, fluorescence is created in the sensor. With a pulse frequency of 10 kHz, the waveform generator (TGA1240, Thurlby Thandar Instruments (TTi) Ltd., Huntington, UK) powers the LED.

A mass flow controller (Model GFC 17, Aalborg Instruments and Controls Inc., New York, NY, USA) precisely controls the concentrations of nitrogen and ammonia in the gas stream. The sample room is completely isolated to avoid interference from outside light sources. A USB 4000 spectrometer (Ocean Optics) is used to detect fluorescence emissions, and a UV-VIS spectrophotometer is used to evaluate fluorophore absorption spectra.

## 3. Results and Discussion

### 3.1. Optical Characterization of the Material Used

**Figure 3:**
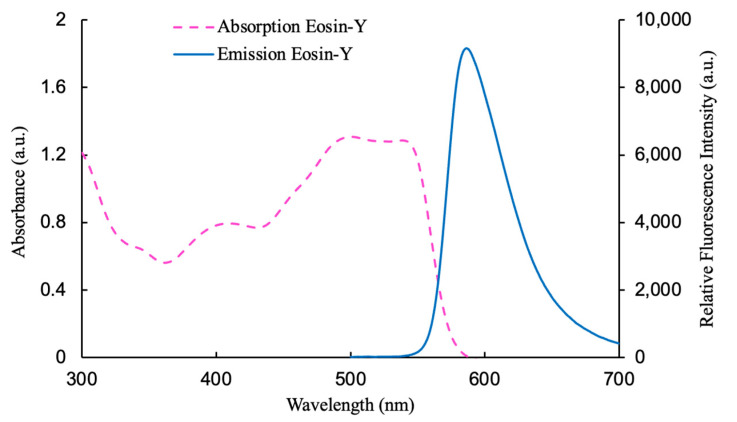
Spectral properties of the material utilized in the optical ammonia sensor, including the absorption spectrum and the emission spectrum of Eosin-Y.

The emission and absorption spectra of the fluorescent dye Eosin-Y are shown in Figure 3. A filter paper was used to measure each chemical absorption and emission spectra with a thickness of 0.20 mm. According to the absorption spectrum, an LED with a core wavelength of 380 nm can efficiently excite all fluorophores. As the ammonia indicator, Eosin-Y exhibits a clear and distinct emission spectrum. The fluorescence emission spectra show peaks between 560 and 580 nm when activated by a 380 nm LED light source (Figure 3). Consequently, each gas is detected through comprehensive monitoring of the emission from the sensing material.

### 3.2. Ammonia Sensing Properties of Single Fiber

**Figure 4:**
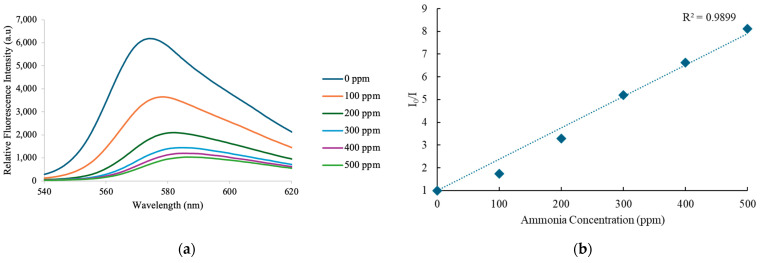
(**a**) Emission spectra of the optical sensor at varying ammonia concentrations and (**b**) Stern–Volmer plots illustrating the ammonia sensing performance.

After conducting the gas sensing experiment, the wavelength shift and linearity observed during the electrospinning process showed increased deviation from the initial condition. As the ammonia content rose from 0 ppm to 500 ppm, exposure to ammonia gas at 580 nm resulted in a progressive decrease in the emission peak, as seen in Figure 4a. When a 380 nm LED light source is used to illuminate the single-fiber sensor, the results clearly show a steady trend of wavelength shifts corresponding to greater ammonia concentrations. These findings confirm that a single sensing device can effectively be an ammonia-sensitive sensor.

The impact of ammonia gas on the ammonia sensing signal’s emission peak is depicted in Figure 4b. The sensor’s exceptional sensitivity is demonstrated by the linearity of the Stern–Volmer (S-V) curve. However, certain aspects exhibit a degree of nonlinearity. The Stern–Volmer plot in Figure 4b exhibits slight deviations from linearity, which may be caused by several factors, such as the non-uniform distribution of the luminophore, where the fluorescence emission within the sensor is not evenly distributed throughout the sensor matrix. This non-uniformity can lead to variations in the interaction between the luminophore and ammonia molecules, making some parts of the sensor more responsive to the gas than others. Another factor is the saturation effect at high ammonia concentrations, causing further increases in ammonia concentration to no longer result in proportional changes in fluorescence intensity. At higher concentrations, the active sites on the luminophore may become saturated, leading to smaller changes in intensity than predicted by the linear model. According to the Stern–Volmer equation, under ideal circumstances with a uniform luminophore environment, several factors influence the quenching of the luminophore in the optical ammonia sensor [41].

*I*_0_/*I* = 1 + *K*_*sv*_ [*NH*_3_] (1)

The Stern–Volmer plot of *I*_0_/*I* versus ammonia gas concentration demonstrates the effectiveness of Eosin-Y as a sensitive material for detecting ammonia. In this context, *I*_0_ represents the luminescence intensity without ammonia, while *I* represents the intensity when ammonia is present. The slope of the plot indicates the Stern–Volmer quenching constant (*Ksv*), with [*NH*_3_] denoting the concentration of ammonia. Ideally, the plot should be linear, with the slope reflecting the quenching dynamics governed by *Ksv*. As shown in Figure 4b, Eosin-Y displayed a maximum sensitivity of 8.11 when exposed to 500 ppm of ammonia. These results confirm the reliability of the device as a single-sensor ammonia probe.

### 3.3. Wavelength Shift of Ammonia-Sensitive Dye

**Figure 5:**
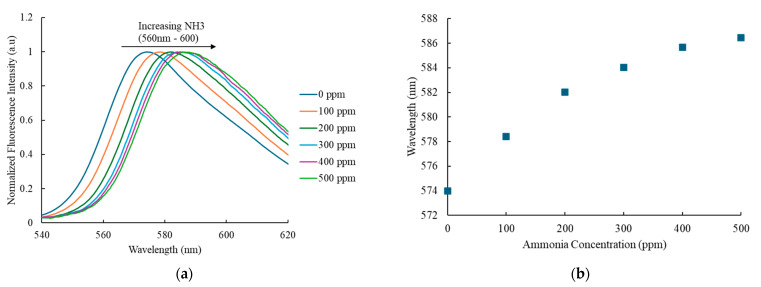
(**a**) Wavelength shifts of the ammonia-sensitive dye and (**b**) a plot showing the wavelength shifts in ammonia sensing.

As ammonia is exposed to ammonia gas concentrations ranging from 0 to 500 ppm, Figure 5a displays the fluorescence intensity normalized by the wavelength shift, demonstrating the gradual change in the optical detecting wavelength. The graph reveals a trend of decreasing peak intensity after exposure to ammonia, along with a shift in the wavelength of Eosin-Y. The wavelength shift that results is more noticeable as the ammonia gas valve is opened to reach the required concentration, which ranges from 0 ppm to 500 ppm.

The correlation between the wavelength shift and rising ammonia gas concentration is shown in Figure 5b. The data indicate that higher ammonia concentrations lead to a more significant wavelength shift. Specifically, the observed wavelength shift is 14.49 nm, with a shift rate of 0.029 nm/ppm relative to the changes in ammonia concentration.

### 3.4. Photostability of Optical Ammonia Sensor

**Figure 6:**
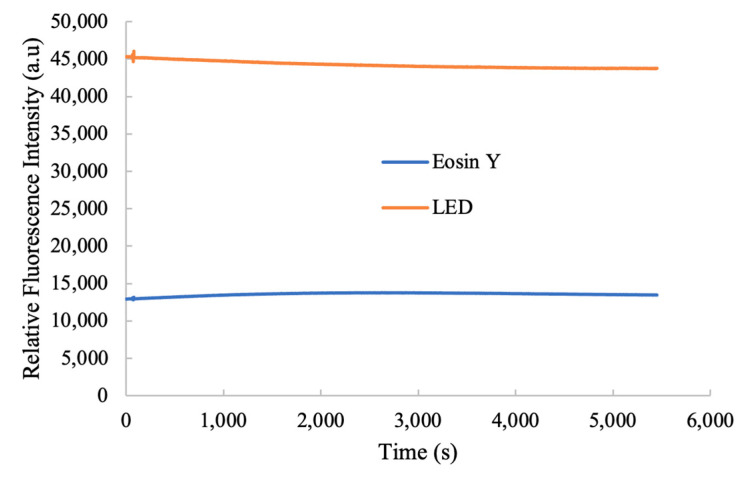
Photostability performance of the optical ammonia sensor.

Eosin-Y is utilized as a reference signal incorporated into a cellulose acetate matrix, which is then applied to a single-fiber ammonia sensor. Figure 6 demonstrates the ammonia photostability of the proposed sensor. An LED provided the illumination with a center peak wavelength of 380 nm. The fluorescence intensity of the ammonia sensor signal on the Eosin-Y-loaded electrospun fiber membrane stayed constant with the reference signal at 580 nm following an extra hour of continuous exposure at ambient temperature. Figure 6 highlights the ammonia photostability of the sensor, with the Eosin-Y-containing electrospun fiber maintaining a consistent reference signal.

### 3.5. Dynamic Response Time of Optical Ammonia Sensors

**Figure 7:**
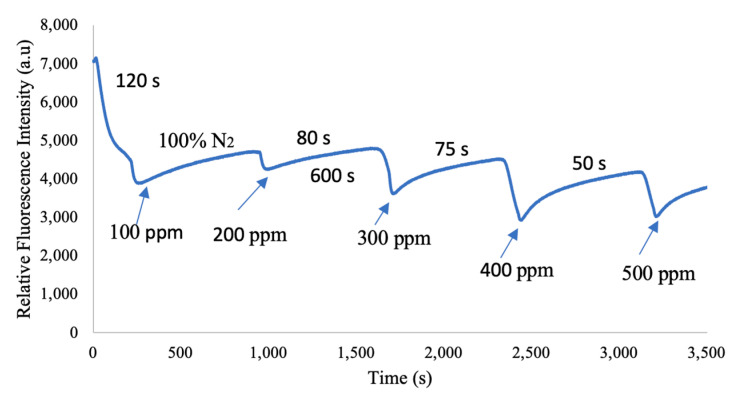
Dynamic response time of the optical ammonia sensor.

The Eosin-Y-based dynamic response and recovery data from the ammonia gas sensor showed a pattern comparable to what was seen in the sensor itself. The dynamic response and recovery behavior of the ammonia gas sensor are depicted in Figure 7. The optical ammonia sensor showed a typical dynamic response as it alternated between 100% nitrogen gas and ammonia gas at concentrations of 100 ppm, 200 ppm, 300 ppm, 400 ppm, and 500 ppm throughout the experiment. The optical ammonia sensor had a 120 s response time when switching from nitrogen to ammonia and a 600 s recovery time when switching back to nitrogen from ammonia.

The response time is a critical factor for optical gas sensing applications. In the first and second cycles, the response times were 120 s and 160 s, respectively, while the recovery time in the first cycle was around 900 s (approximately 15 min). A notable issue arose with the recovery time after switching from 500 ppm ammonia to 100% nitrogen, as the output stayed elevated for more than 15 min. However, it was observed that ammonia sensing could quickly revert to the initial peak signal when nitrogen was used as a neutralizer. The long recovery time of optical ammonia (NH_3_) sensors is due to strong NH_3_ adsorption, slow diffusion, weak optical response reversibility, and limited airflow. NH_3_ forms strong bonds with materials like metal oxides and polymers, making desorption slow, especially in thick or dense sensing layers. Environmental factors like humidity further delay recovery by competing for adsorption sites.

To improve the response time of an Eosin-Y-doped electrospun fiber optical sensor for ammonia (NH_3_) detection via wavelength shifts, optimizing fiber morphology is crucial. Reducing fiber diameter and increasing porosity enhances gas diffusion, while controlling Eosin-Y loading prevents aggregation and ensures a uniform optical response. Structuring fiber alignment further optimizes light transmission, enhancing response speed. These strategies enable faster and more reliable ammonia detection, improving the sensor’s dynamic performance.

### 3.6. Effect of Temperature and Humidity of Optical Ammonia Sensor

**Figure 8:**
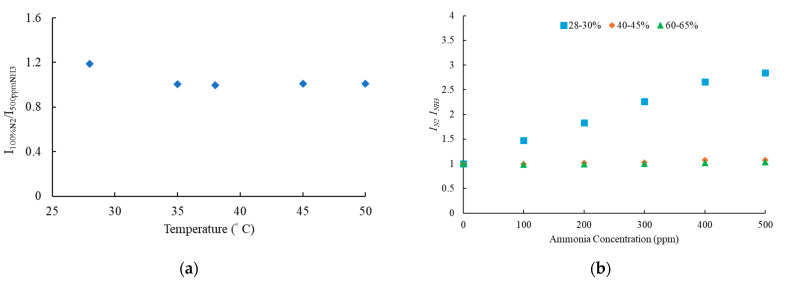
(**a**) Temperature impact on the sensitivity and (**b**) humidity of ammonia.

Ammonia sensitivity should be evaluated at different temperatures to assess sensor performance. The impact of temperature on the optical ammonia sensor during measurement is shown in Figure 8a. The sensitivity and intensity of the ammonia-sensitive dyes decrease at the first temperature. However, the ammonia sensor will stabilize on a long-term temperature effect.

Additionally, ammonia-sensitive dyes should be tested under varying humidity levels to evaluate their sensing capability in different environmental conditions. Figure 8b shows the impact of humidity on the ammonia sensor. Changes in humidity influence ammonia sensitivity, with lower humidity levels and room temperature or humidity-controlled conditions enhancing sensor sensitivity. In contrast, high humidity levels tend to reduce sensor performance.

### 3.7. Selectivity of Optical Ammonia Sensor

**Figure 9:**
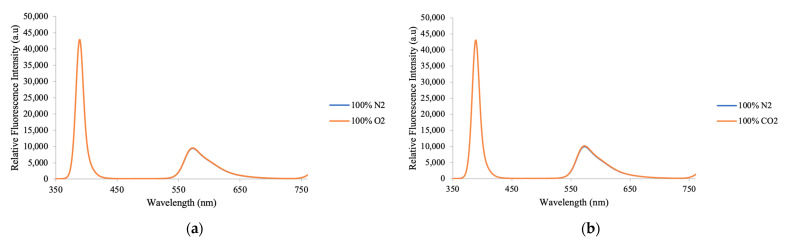
The optical ammonia sensor exhibits a response to (**a**) oxygen and (**b**) carbon dioxide.

Under rare experimental circumstances, too much gas can occasionally prevent a gas sensor from operating as intended. As a result, the gas sensor’s selectivity needs to be considered. This test evaluates the ammonia sensor’s response to oxygen and carbon dioxide. For 30 min, the ammonia sensor is alternately exposed to oxygen and carbon dioxide gas to measure any variations in fluorescence intensity. The results obtained under 100% oxygen are shown in Figure 9a, where no significant change in fluorescence intensity is observed. Similarly, there is no variation in intensity when exposed to carbon dioxide gas. These findings confirm that the proposed ammonia sensor is insensitive to oxygen and carbon dioxide gas concentrations.

## 4. Conclusions

A wavelength shift is seen in this study, where an increase in ammonia concentration results in a decrease in the fluorescence intensity at 580 nm. Eosin-Y, a fluorescent dye, was used in the electrospinning process to create a single-fiber sensor for ammonia gas detection successfully. The experimental findings reveal that the sensor demonstrates excellent sensitivity and linearity, with a wavelength shift sensitivity of 0.029 nm/ppm in response to varying ammonia concentrations. Furthermore, the ammonia-sensitive dyes displayed strong selectivity, showing no interference when exposed to other gases such as oxygen and carbon dioxide. Overall, the electrospun fiber developed in this research provides an efficient solution for detecting ammonia concentrations. Finally, fabricating an optical sensor based on Eosin-Y-doped electrospun fibers for ammonia detection should focus on enhancing the sensor’s robustness and reusability in real-world conditions. Exploring the development of advanced regeneration techniques and protective coatings and optimizing the sensor material could improve its long-term performance. Additionally, investigating the sensor’s integration with portable detection systems for continuous monitoring and its adaptability for a broader range of gases will be valuable for broadening its applications in environmental monitoring, industrial safety, and medical diagnostics.

## Data Availability

Data are contained within the article.
